# Shaving the Beard

**Published:** 1854-05

**Authors:** 


### HALL'S JOURNAL OF HEALTH.

VOL. I.] MAY, 1854. [NO. V.

### SHAVING THE BEARD.

(From "*The Boston Medical and Surgical Journal.*)

The more I reflect upon the mysteries of neurology and animal chemistry, the more confident I am that, while we are the least suspecting it, trifling errors in our daily life are producing important effects upon our corporeal systems; and I declare it as my deliberate conviction, that the habit, which may almost be styled American, of using the razor upon the face, is sufficient to cause a large proportion of the lamentable evils which affect the human race in this country.

It appears by experiment that the beard, if shaved, grows four to five times faster than if unshorn. In this calculation, an item is omitted which it is difficult to estimate, *i. e.*, the stimulus given the beard, by the first application of the razor in adolescence, the experiments being made upon beards after they have acquired an unnaturally rapid growth. The effect of this early stimulus may be fairly counted at double the natural growth; then reckoning the difference in size and weight of the fibre, which is treble, and we find the frightful truth to be, that we raise thirty times the natural quantity of beard! Thus it is evident that the true beard is exhausted at a very early age, after which the system is forced to supply a substitute. Now nature will not submit with impunity to extraordinary demands upon her vigor, and that which requires her to produce in a lifetime thirty times as much beard as she was first inclined to, must certainly be considered as such. She is fatigued in proportion to the effort, let the particular kind be what it may; although her recuperative powers are great, she insists upon having repose, even when working at a rate chosen by herself. If that repose is denied her, she takes her revenge by breaking down the mechanism. Who then, can estimate the revenge she will take for being compelled to labor without rest under an uncompromising task-master ?

2d. The chemical laboratory of man furnishes in just proportion the ingredients required to deposite in suitable quantity the bones, skin, hair, nails, &c., and it is obvious that a superstraining of those chemical elements which enter into the composition of the beard must deprive of their just due all the other tissues which are wholly or in part composed of the same elements. Such injustice to other structures they must inevitably feel, and the entire system must suffer from a disturbance of the balance of power requisite to a healthy action of its various parts.

3d. The proper calorification of the body is one of the most essential conditions of its healthy action; and the non-conducting properties of the beard ought to be a caution against trifling with so powerful an agent, more especially when one considers its intimate connection with the calorific organs of the brain and with the respiratory organs. The popular notion, that, as women are beardless, men may be or not as they please, is founded in misapprehension. A man and a woman form one specimen of the *genus homo*, and from a physiological point of view must be considered one and the same. The absence of beard in the woman is countervailed by some other differences in her constitution, which it would be needless to point out even if we knew them. It suffices to know that nature is perfect in her work.

4th. The errors of the father shall be visited upon the children unto the third and fourth generation, the tree being known by its fruit, for a corrupt tree cannot bring forth good fruit; which, simplified, is, "like begets like." No person who feels the force of this law in all its fulness, can expect to transmit to his posterity vigorous pulmonary organs, if he has done the best he could to ruin his own. Daughters and sons are by nature equally their father's heirs, and if consumption of the respiratory organs spares more men than women, the out-door exercise of men must in part account for the difference.

The mania which has ever possessed man for disfiguring himself is astonishing. Not satisfied with God's most perfect handiwork, different tribes and nations variously undertake to beautify it, thus fairly making themselves laughing stocks for each other; but it is to be hoped that the "pioneers of civilization will come out from the category of those who tattoo the skin, flatten the skull, shave the crown, taper the waist, stint the feet, circumcise, and slit their ears and noses.

It is with difficulty that old habits are renounced, even when one is convinced that life can be prolonged and made happier thereby; but it is a question for young men seriously to consider, whether, on starting in life, they will addict themselves to a habit which at once wastes the time, sours the temper, is against nature, and, consequently, involves their health, and that of their offspring.

Nature has made her terms with us how we may enjoy our daily existence and lengthen out our lives; these terms are—to know her laws and not infringe them.

